# Translational Research to Improve the Efficacy of Carbon Ion Radiotherapy: Experience of Gunma University

**DOI:** 10.3389/fonc.2016.00139

**Published:** 2016-06-09

**Authors:** Takahiro Oike, Hiro Sato, Shin-ei Noda, Takashi Nakano

**Affiliations:** ^1^Department of Radiation Oncology, Gunma University Graduate School of Medicine, Gunma, Japan; ^2^Gunma University Heavy Ion Medical Center, Gunma, Japan

**Keywords:** carbon ion radiotherapy, patient selection, combination therapy, translational research, treatment planning

## Abstract

Carbon ion radiotherapy holds great promise for cancer therapy. Clinical data show that carbon ion radiotherapy is an effective treatment for tumors that are resistant to X-ray radiotherapy. Since 1994 in Japan, the National Institute of Radiological Sciences has been heading the development of carbon ion radiotherapy using the Heavy Ion Medical Accelerator in Chiba. The Gunma University Heavy Ion Medical Center (GHMC) was established in the year 2006 as a proof-of-principle institute for carbon ion radiotherapy with a view to facilitating the worldwide spread of compact accelerator systems. Along with the management of more than 1900 cancer patients to date, GHMC engages in translational research to improve the treatment efficacy of carbon ion radiotherapy. Research aimed at guiding patient selection is of utmost importance for making the most of carbon ion radiotherapy, which is an extremely limited medical resource. Intratumoral oxygen levels, radiation-induced cellular apoptosis, the capacity to repair DNA double-strand breaks, and the mutational status of tumor protein p53 and epidermal growth factor receptor genes are all associated with X-ray sensitivity. Assays for these factors are useful in the identification of X-ray-resistant tumors for which carbon ion radiotherapy would be beneficial. Research aimed at optimizing treatments based on carbon ion radiotherapy is also important. This includes assessment of dose fractionation, normal tissue toxicity, tumor cell motility, and bystander effects. Furthermore, the efficacy of carbon ion radiotherapy will likely be enhanced by research into combined treatment with other modalities such as chemotherapy. Several clinically available chemotherapeutic drugs (carboplatin, paclitaxel, and etoposide) and drugs at the developmental stage (Wee-1 and heat shock protein 90 inhibitors) show a sensitizing effect on tumor cells treated with carbon ions. Additionally, the efficacy of carbon ion radiotherapy can be improved by combining it with cancer immunotherapy. Clinical validation of preclinical findings is necessary to further improve the treatment efficacy of carbon ion radiotherapy.

## Introduction

Carbon ion radiotherapy holds great promise for cancer therapy. Carbon ions have two advantages over X-rays such as a sharp dose distribution and a strong cell-killing capacity (1). Clinical trials show that carbon ion radiotherapy has excellent antitumor effects (2, 3). Moreover, it is suggested that carbon ion radiotherapy is an effective treatment for tumors that are resistant to conventional X-ray radiotherapy (4–6).

The National Institute of Radiological Sciences (NIRS) initiated carbon ion radiotherapy in Japan in the year 1994 using the Heavy Ion Medical Accelerator in Chiba (HIMAC). Up until January 2016, more than 8000 patients with different types of cancer were treated at the NIRS. The excellent clinical outcomes encouraged widespread use of carbon ion radiotherapy (2). However, the high cost of constructing the accelerator system limits its practical application. New accelerator systems were designed to overcome this limitation; as a result, the cost of the accelerator systems was reduced to approximately $100,000,000 that accounts for one-third of the corresponding HIMAC parameters. Gunma University Heavy Ion Medical Center (GHMC) was launched in the year 2006 as a proof-of-principle institute for carbon ion radiotherapy based on the newly introduced compact accelerator systems. GHMC commenced operation of the accelerator systems in 2009 and performed the first carbon ion radiotherapy for cancer in 2010. All carbon ion radiotherapy carried out at GHMC has been performed as prospective clinical trial (Table 1). As of January 2016, GHMC has treated more than 1900 cancer patients with carbon ion radiotherapy, without any major incidents.

**Table 1:** **Clinical trails on carbon ion radiotherapy at GHMC**.

Trial ID	Patient enrollment	Cancer type	Major indication criteria	Total dose/fr.	Combined therapy
Gunma0905	2012-	Cranial base tumor	No CNS invasion	60.8 GyRBE/16 fr.	–
Gunma0901	2010–2013	H&N cancer (except Sq, melanoma, sarcoma)	N0/1M0	57.6 or 64 GyRBE/16 fr.	–
Gunma0902	2012-	H&N musculoskeletal tumor	N0/1M0	70 GyRBE/16 fr.	–
Gunma0903	2012-	H&N melanoma	N0M0	57.6 or 64 GyRBE/16 fr.	Concurrent DTIC, ACNU, and VCR
Gunma0701	2010–2015	NSCLC	Stage I, peripheral, inoperable	60 GyRBE/4 fr.	–
Gunma1201	2013–2015	NSCLC	Stage III, inoperable	40 GyRBE/10 fr. (ENI) + 20 or 24 GyRBE/6 fr. (IFI)	–
Gunma0703	2010–2013	Hepatcellular carcinoma	Tumor diameter ≤10 cm	52.8 GyRBE/4 fr.	–
Gunma1203	2013	Hepatcellular carcinoma	Tumor diameter 3–10 cm	60 GyRBE/4 fr.	–
Gunma1303	2013–2015	Hepatcellular carcinoma	Adjacent to digestive tract	60 or 64.8 GyRBE/12 fr.	–
Gunma1301	2013–2015	Pancreatic cancer	T4N0/1, inoperable	52.8 or 55.2 GyRBE/12 fr.	Concurrent gemcitabine
Gunma1501	2015-	Pancreatic cancer	T4N0/1M0	55.2 GyRBE/12 fr.	Concurrent S-1
Gunma0801	2010-	Rectal cancer	Postoperative local recurrence	73.6 GyRBE/16 fr.	–
Gunma0702	2010–2013	Prostate cancer	≤T3	57.6 GyRBE/16 fr.	± Hormone therapy
Gunma1302	2013-	Prostate cancer	≤T3	57.6 GyRBE/16 fr.	± Hormone therapy
Gunma1103	2013-	Prostate cancer	Castration resistant cancer	57.6 GyRBE/16 fr.	± Hormone therapy
Gunma1202	2013–2014	Uterine cervical cancer	Locally advanced	36 GyRBE/12 fr. (WPI) + 19.2 GyRBE/4 fr. (IFI)	Concurrent CDDP followed by ICBT
Gunma1401	2014-	Uterine cervical cancer	Locally advanced	36 GyRBE/12 fr. (WPI) + 19.2 GyRBE/4 fr. (IFI)	Concurrent CDDP followed by ICBT
Gunma0904	2010–2013	Musculoskeletal tumors	N0M0	64, 67.2, or 70.4 GyRBE/16 fr.	–
Gunma1102	2011–2013	Musculoskeletal tumors (pediatric)	Age 6–16, inoperable	60.8, 64, 67.2, or 70.4 GyRBE/16 fr.	–
Gunma1101	2011-	Lymph node metastatic tumor	1–3 nodes in 1 irradiation field	48 or 52.8 GyRBE/12 fr.	–
Gunma1304	2013-	In-field recurrent tumor	previously treated by radiotherapy	Various according to disease site	–
Gunma1204	2013-	Tumor resistant to standard Tx	known to be resistant to standard Tx	Various according to disease site	–

*fr., fractions; CNS, central nervous system; H&N, head and neck; Sq, squamous cell carcinoma; NSCLC, non-small cell lung carcinoma; DTIC, dacarbazine; ACNU, nimustine; VCR, vincristine; ENI, elective nodal irradiation; IFI, involved field irradiation; WPI, whole pelvic irradiation; CDDP, cisplatin; ICBT, intracavitary brachytherapy; Tx, therapy*.

Along with carbon ion radiotherapy, GHMC engages in translational research to improve the efficacy of this treatment modality with financial support from the Japan Society for the Promotion of Science through its two umbrella programs: the Twenty-First Century Centers of Excellence Program (2004–2008) and the Strategic Young Researcher Overseas Visits Program for Accelerating Brain Circulation (2013–2016). Translational research at GHMC was further accelerated by establishment of the Gunma University Initiative for Advanced Research in 2015, in which the Department of Radiation Oncology of the Massachusetts General Hospital/Harvard Medical School launched a Japanese branch to stimulate interdisciplinary collaboration in the field of heavy ion radiation biology. In addition, GHMC contributes to the education and development of global leaders in the field of heavy ion radiation therapy through the Program for Cultivating Global Leaders in Heavy Ion Therapeutics and Engineering, supported by the Japanese Ministry of Education, Culture, Sports, Science, and Technology (2012–2018). Here, we summarize achievements in translational research on carbon ion radiotherapy performed at GHMC through these scientific endeavors.

## Research to Guide the Selection of Patients Suitable for Carbon Ion Radiotherapy

The number of newly diagnosed cancer patients worldwide is ~14 million/year (7). By contrast, the maximum number of patients that can be treated by carbon ion radiotherapy worldwide is estimated to be ~2100/year (Table 2) (8). Thus, carbon ion radiotherapy has the capacity to treat only 0.015% of the total patient population with a newly diagnosed cancer. Moreover, even in Japan, which has the highest density of facilities for carbon ion radiotherapy in the world, carbon ion radiotherapy has the capacity to treat only 0.20% of newly diagnosed cancer cases. These facts highlight the extremely limited availability of this medical resource. Although 11 facilities for carbon ion radiotherapy are currently under construction or are planned for construction (1), the critical shortage of facilities will not be resolved in any practical way for a few decades. Therefore, selecting patients who can derive the greatest benefit from carbon ion radiotherapy is of great importance. Early clinical experience shows that carbon ion radiotherapy is an effective treatment for tumors that are resistant to conventional X-ray radiotherapy (4–6); therefore, carbon ion radiotherapy will be the most beneficial for patients with these types of tumor. From this point of view, assays that predict the X-ray sensitivity of a tumor are urgently required to facilitate appropriate selection of patients for carbon ion radiotherapy.

**Table 2:** **Number of cancer patients treated by carbon ion radiotherapy worldwide per year**.

S. No.	Country	City	Facility	Case/year	Year
1	Japan	Chiba	NIRS	888	2013
2	Japan	Gunma	GHMC	448	2013
3	Japan	Hyogo	HIBMC	270	2013
4	Japan	Saga	HIMAT	132	2013
5	China	Lanzhou	HIRFL	27	2006–2013 in average
6	Germany	Heidelberg	HIT	274	2009–2013 in average
7	Italy	Pavia	CNAO	53	2012–2013 in average

*NIRS, National Institute of Radiological Sciences; GHMC, Gunma University Heavy Ion Medical Center; HIBMC, Hyogo Ion-Beam Medical Center; HIMAT, Heavy Ion Medical Accelerator in Tosu; HIRFL, Heavy Ion Research Facility in Lanzhou; HIT, Heidelberg Ion-Beam Therapy Center; CNAO, Centro Nazionale Adroterapia Oncologica*.

*Data on facility #1–4 are based on the website of Association for Nuclear Technology in Medicine (written in Japanese): http://www.antm.or.jp/05_treatment/01.html. Data on facility #5–7 are based on Ref. (8)*.

Histopathological typing of tumors is performed to predict treatment responses in the clinical setting of X-ray radiotherapy. Nevertheless, the response varies widely according to tumor type, and even among those with the same histological type. Thus, additional indices that support prediction of X-ray sensitivity according to histopathological type are required. For many types of cancer, the SF2 value, i.e., the surviving fraction of X-irradiated tumor cells (irradiated *ex vivo* with a dose of 2 Gy) measured in a clonogenic survival assay, correlates with clinical outcome of X-ray radiotherapy (9). However, the SF2 value has shortcomings, i.e., primary culture of the tumor cells required for the clonogenic assay is difficult, and necessitates 2 weeks to obtain final results. Therefore, the SF2 value is not widely used in the clinic. Previously, we identified several cellular mechanisms that contribute to the resistance of cancer cells to X-rays, including intratumoral hypoxia, resistance to radiation-induced apoptosis, a high capacity for the repair of DNA double-strand breaks (DSBs), and mutations in certain oncogene and tumor suppressor genes. By focusing on these factors, we propose the following predictive assays for determining the X-ray sensitivity of cancer cells.

**Figure 1:**
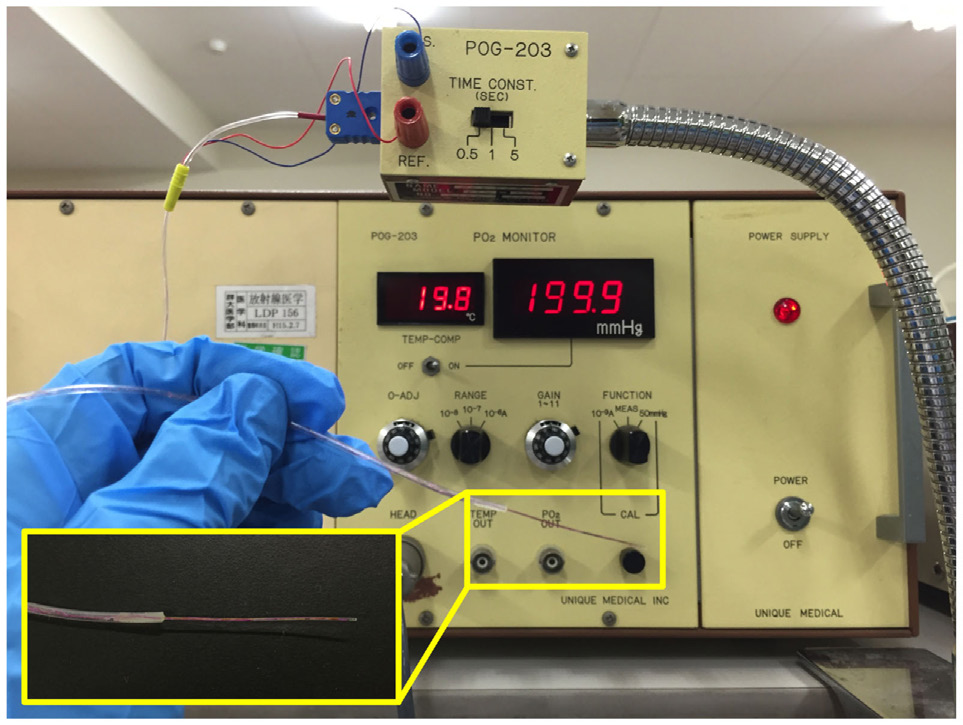
**Tools for intratumoral pO_2_ measurement**. A needle-type polarographic oxygen electrode is used by direct insertion into a tumor.

**Figure 2:**
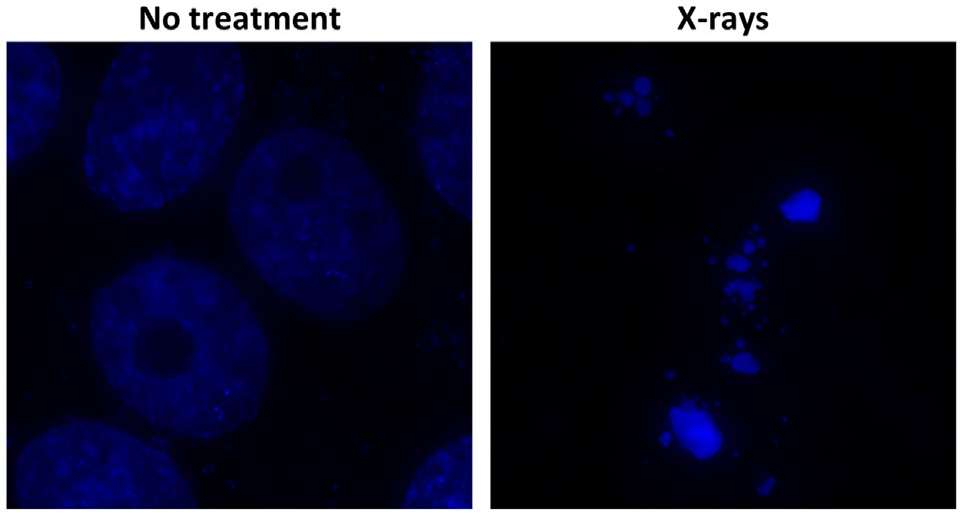
**Radiation-induced apoptosis, as assessed by DAPI staining**. Cultured Ma-24 lung cancer cells were stained with DAPI at 72 h after irradiation using X-rays at a dose of 4 Gy. Apoptotic cells are identified by the appearance of apoptotic bodies, characterized by condensed and fragmented nuclei, under a fluorescence microscope.

Intratumoral hypoxia is a major contributor to the X-ray resistance of cancer cells (10–12). Nakano et al. used a needle-type polarographic oxygen electrode to measure intratumoral oxygen partial pressure (pO_2_) in patients with locally advanced uterine cervical cancer treated using X-ray radiotherapy (13) (Figure 1). The authors found that low pretreatment intratumoral pO_2_ values correlated with poor outcomes after X-ray radiotherapy. On the other hand, carbon ion radiotherapy showed good antitumor effects in patients with locally advanced uterine cervical cancer, irrespective of pretreatment intratumoral pO_2_ levels. These data indicate that assays to determine pretreatment intratumoral pO_2_ values will be useful for identification of X-ray-resistant tumors profiting from carbon ion radiotherapy. Importantly, recent studies indicate that as many as 50% of tumors have hypoxic regions, which could underpin X-ray treatment failure and expand the indications for carbon ion radiotherapy (14). Cancer cell resistance to radiation-induced apoptosis is another major factor that contributes to X-ray resistance. Preclinical studies suggest that carbon ions effectively kill cancer cells that are resistant to apoptosis induced by X-ray irradiation (15, 16). Another mode of clonogenic cell death, called mitotic catastrophe and necrosis, is involved in efficient killing of apoptosis-resistant cancer cells by carbon ions (15, 16). Apoptosis following irradiation is readily assessed by morphological observation of nuclei stained with 4′,6-diamidino-2-phenylindole dihydrochloride (DAPI) (Figure 2). Amornwichet et al. demonstrated that apoptosis in HCT116 colon cancer cells peaked at 72 h post-X-ray irradiation, as assessed by DAPI staining (16). This is consistent with the observation that radiation-induced apoptosis in solid tumors mainly corresponds to the so-called late apoptosis, which occurs a few days post-irradiation (17). Furthermore, the DAPI-based assay is easier and faster to perform than the clonogenic survival assay used to calculate the SF2 value. Therefore, DAPI staining of *ex vivo*-irradiated tumor specimens at 72 h post-irradiation is useful for identifying tumors that are resistant to X-ray-induced apoptosis and would therefore benefit from carbon ion radiotherapy.

**Figure 3:**
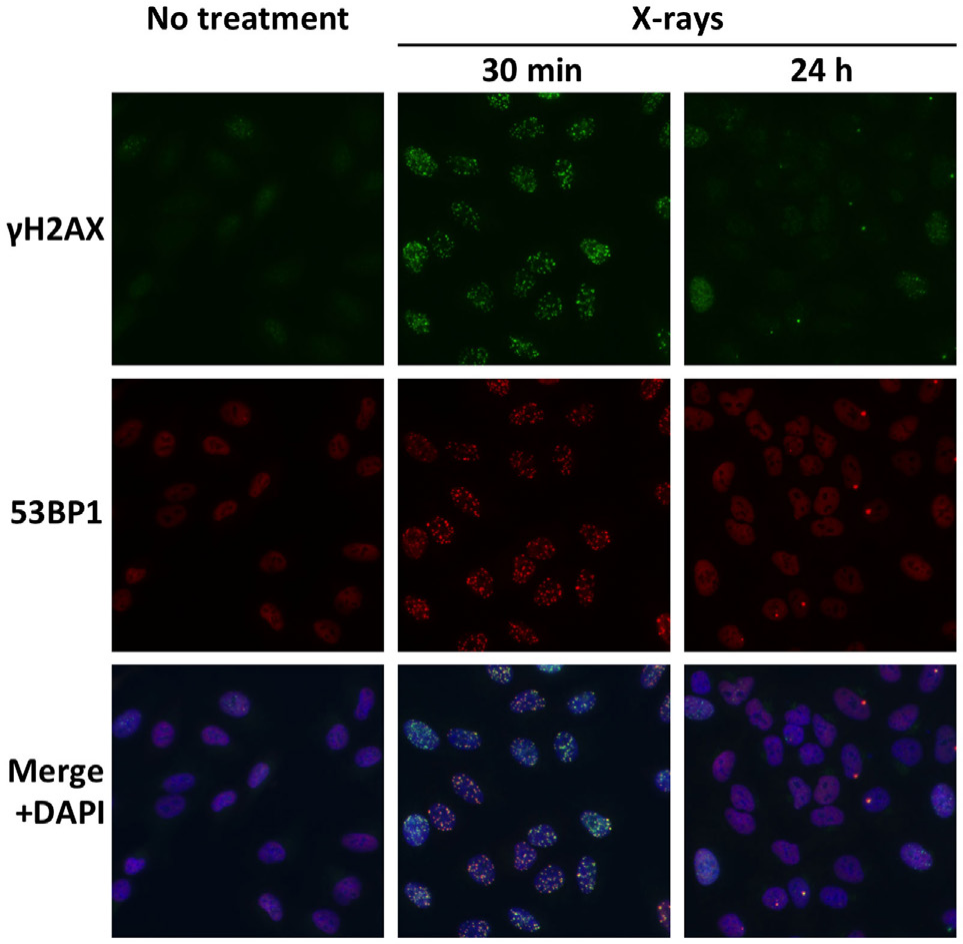
**Radiation-induced DSBs visualized by immunofluorescence staining of γH2AX and 53BP1**. Cultured A549 lung cancer cells were immunostained for γH2AX and 53BP1 at 30 min or 24 h post-irradiation using X-rays at a dose of 1 Gy. DSBs are identified as foci of γH2AX and 53BP1. Merged images show high consistency between γH2AX foci and 53BP1 foci. A markedly smaller number of γH2AX and 53BP1 foci at 24 h compared with 30 min indicate the high capacity of the X-ray-resistant cell line for DSB repair.

Double-strand breaks are major cytotoxic lesions that cause cancer cell death after exposure to ionizing radiation (17). Preclinical studies indicate that the high capacity of cancer cells for DSB repair contributes to X-ray resistance (18, 19). Meanwhile, the cell-killing actions of carbon ions are less affected by intrinsic DSB repair capacity (18, 19). Most likely, complex carbon ion-induced DSBs are more difficult to repair than X-ray-induced DSBs; these persistent unrepaired DSBs then lead to mitotic catastrophe (16). These data indicate that tumors with a high capacity for DSB repair are suitable for carbon ion radiotherapy. DSB repair capacity can be evaluated by immunofluorescence staining for Ser139-phosphorylated histone H2AX (γH2AX) or p53-binding protein 1 (53BP1) because DSBs are detected as foci of γH2AX or 53BP1 (Figure 3) (20–22). The number of foci at 30 min post-irradiation can be used as an index for radiation-induced DSBs. On the other hand, the number of foci in irradiated cells decreases by more than 90% within 24 h post-irradiation, indicating that a major proportion of radiation-induced DSBs is repaired by that time point (20). Thus, the ratio of the foci number at 24 h post-irradiation to that at 30 min post-irradiation can be used as an index for DSB repair capacity. Importantly, the high DSB repair capacity (as indicated by low number of foci at 24 h post-irradiation) is associated with a high rate of clonogenic survival (19). Hence, assay of γH2AX or 53BP1 foci in *ex vivo*-irradiated tumor specimens can be performed to identify tumors with a high capacity for DSB repair and suitable for carbon ion radiotherapy.

Cancer cells harbor modifications in a number of molecular pathways that affect intrinsic radiosensitivity. Mutations in oncogenes and tumor suppressor genes are common, and these mutations result in alterations in signaling pathways. We previously showed that inactivating mutations in the gene encoding tumor suppressor protein 53 (*TP53*) confer X-ray resistance on cancer cells (15, 16, 23). We also showed that epidermal growth factor receptor gene (*EGFR*) mutation-negative non-small cell lung cancer (NSCLC) cells are more resistant to X-rays than *EGFR* mutation-positive NSCLC cells (19). These findings were validated by clinical studies (24–27). Interestingly, investigations using isogenic cancer cell lines demonstrated that carbon ions can kill cancer cells irrespective of the mutational status of *TP53* and *EGFR* (15, 16, 19, 23). Taken together, these data indicate that the mutational status of *TP53*/*EGFR* is useful for selecting patients who are suited for carbon ion radiotherapy. Nevertheless, a recent genome-wide analysis revealed the presence of hundreds of gene mutations in a single tumor (28). Because the overall radiosensitivity of a tumor should be the result of this highly complex genetic context, the mutational status of only a small subset of well-known cancer-related genes (e.g., *TP53* and *EGFR*) may not be the best predictor of radiosensitivity. Thus, studies aimed at elucidating detailed gene mutation profiles to facilitate better prediction of tumor radiosensitivity are warranted.

## Research Aimed at Optimizing Carbon Ion Radiotherapy

Optimization of carbon ion radiotherapy can be addressed using two approaches such as radiation physics and radiation biology. Both physics and biology play intertwining roles in treatment planning; therefore, advances in one field benefit the other. For example, increased irradiation accuracy results in less normal tissue toxicity. By contrast, accurate information about the biological characteristics of tumors and normal tissues aids optimal treatment planning. Biological factors that affect the treatment procedure, including biological responses to dose fractionation, normal tissue toxicity, tumor cell motility, and the bystander effect, are discussed below.

In X-ray radiotherapy, the rationale for dose fractionation is provided by the re-oxygenation and cell cycle redistribution of tumor cells, as well as a higher capacity for the repair of sublethal damage in normal tissues versus tumors (17). The cell-killing effect of carbon ions versus X-rays is less dependent on these factors (13, 17); therefore, the responses of tumors and normal tissues to carbon ions may be different from those to X-rays, even when the same dose fractionation schedule is utilized. To address this issue, Ando et al. used a mouse model to explore the effects of carbon ion dose fractionation on tumor and normal tissues (29). The investigators treated fibrosarcoma xenografts and host mouse skin with γ rays or carbon ion beams with three different linear energy transfer (LET) values (20, 42, and 77 keV/μm) and with different fractionation schedules (i.e., one to seven fractions). Interestingly, the relative biological effectiveness (RBE) values for tumor growth delay were higher than those for early skin reaction when 42- and 77-keV/μm carbon ion beams, but not γ rays or 20-keV/μm carbon ion beams, were employed in intermediate fractionation schedules (i.e., two to six fractions). The therapeutic gain (calculated as the ratio of the RBE value for tumor growth delay to that for early skin reaction) was maximized for the 42 keV/μm beams delivered in four fractions. Yoshida et al. examined the impact of carbon ion dose fractionation on the small intestine by assessing crypt survival in the mouse model employed above (30). In contrast to the results for early skin reaction, no therapeutic gain was observed for the intermediate fractionation schedules. This might be because intestinal crypt cells have a low capacity to repair sublethal damage induced by carbon ions. These two studies indicate that different strategies are required to optimize the dose fractionation schedules used for carbon ion radiotherapy in the skin versus the small intestine. With respect to the skin, the therapeutic window for carbon ion irradiation can be expanded by employing an intermediate hypofractionation strategy. Therefore, the actual fractionation schedule that corresponds to “intermediate” hypofractionation in the mouse model should be further explored in the clinic. On the other hand, the therapeutic window for carbon ions and X-rays in the small intestine may be comparable, and the benefit of dose fractionation may be lower for carbon ions than for X-rays. This indicates that, in abdominal irradiation to treat tumors such as uterine cervical cancer, the maximum tolerable carbon ion dose can be delivered in a smaller number of fractions, resulting in a shorter treatment period. In addition, hypofractionated carbon ion radiotherapy that results in the shorter treatment period compared with X-ray radiotherapy utilizing conventional 2 Gy/day fractionation can contribute to reduce tumor repopulation effect. Assessment of the effect of carbon ion dose fractionation in different tumors and normal tissues in the same mouse model should be further investigated, together with concomitant evaluation of factors that can affect the results of fractionated irradiation (i.e., oxygen levels, cell cycle profiles, and DSB repair capacity).

Carbon ion radiotherapy shows a steep dose fall-off; therefore, the treatment plans are more susceptible to target motion than the plan for three-dimensional conformal radiotherapy (3D-CRT) with X-rays. A larger target volume setting increases the robustness of the dose delivered to the tumor; however, it also increases toxicity to adjacent normal tissues. Therefore, it is necessary to determine the sensitivity of normal tissues to obtain the optimal target volume setting. The nervous system is critically at risk of radiotherapy toxicity, because it is a serial organ with low redundancy and low capacity for regeneration. The sensitivity of the central nervous system to carbon ions has been examined in multiple experimental models. Isono et al. evaluated the sensitivity of human neural stem cells to carbon ions and found that the RBE value, as assessed by cell proliferation, was 2.0 (31). Yoshida et al. examined the effects of carbon ion irradiation in normal rat brain (32). The authors used an organotypic slice culture of cerebellum excised from 10-day-old rats and assessed morphological changes and cellular apoptosis, defined as disorganization of the external granule cell layer and positive staining for TdT-mediated dUTP-biotin nick-end labeling (TUNEL), respectively. They found that the RBE value for rat cerebellum was 1.4–1.5. Kaminuma et al. also explored carbon ion-provoked apoptosis in the rat brain by performing a TUNEL assay in a primary culture of fetal hippocampal neurons (33). The RBE value was strikingly high at 10.2. Similarly, Al-Jahdari and colleagues investigated the sensitivity of the peripheral nervous system to carbon ions by employing dorsal root ganglia and sympathetic ganglion chains prepared from the chick embryo at days 8 and 16, representing the immature and mature peripheral nervous system (34). Growth cone collapse was assessed as an index of malfunction in the neuronal network, yielding an RBE value of 3.1–3.2 in day 8 neurons and 1.5–2.1 in day 16 neurons. Meanwhile, the RBE value assessed by TUNEL staining was 2.5–2.9 in day 8 neurons and 1.4–1.8 in day 16 neurons. Although it is difficult to draw a firm conclusion from the above studies employing different experimental models, nervous systems (central and peripheral) and endpoints, the data collectively indicate that the RBE value of the adult nervous system is ~1.4–2.1 when morphological changes and cellular apoptosis are utilized as endpoints. Given the fact that the RBE value for carbon ions in cancer cells is generally ~2–3, these findings suggest that carbon ion radiotherapy has a wider therapeutic window than X-rays when used to treat tumors adjacent to the components of the central and peripheral nervous systems. Notably, these data also indicate that immature neurons are more sensitive to carbon ion irradiation than mature neurons. Thus, careful attention should be paid to neural toxicity when carbon ion radiotherapy is used to treat pediatric tumors.

The lung is another critical organ at risk in radiotherapy. Radiation-induced lung injury can be lethal in some patients and is a major dose-limiting factor for thoracic irradiation (35). Okano et al. used a crystal violet staining assay to examine the effect of carbon ions on the proliferation of immortalized human small airway epithelial cells (iSAECs) and normal human lung fibroblasts (36). The resultant RBE value was 3.2 for iSAECs and 2.2 for normal lung fibroblasts. On the other hand, ionizing radiation can indirectly damage normal lung tissue by triggering inflammatory reactions. Upregulation of intercellular adhesion molecule-1 (ICAM-1) expression on the surface of pulmonary endothelial cells participates in this inflammation-related process by increasing macrophage infiltration into the lung (37, 38). Kiyohara et al. compared ICAM-1 expression on the surface of human umbilical vein endothelial cells after irradiation with carbon ions and X-rays. The data showed that post-irradiation ICAM-1 expression levels were 2.56- and 2.47-fold higher after carbon ion irradiation than after X-ray irradiation at 1 and 2 Gy, respectively (39). These data signify that the estimated RBE values in normal lung tissue and lung cancer cells are comparable (~2–3) (19).

Several studies demonstrate that X-ray irradiation increases the motility of cancer cells (40, 41). The migration of irradiated cancer cells may influence the setting of target volumes, i.e., the margin from the gross tumor volume (GTV) to the clinical target volume (CTV). Murata et al. used a wound healing assay and F-actin staining to examine the effect of carbon ions on the motility of A549 lung cancer cells (42). Carbon ion irradiation promoted the healing of scratch wounds in cell monolayers and increased the formation of F-actin protrusions, both indicators of increased cancer cell motility. Interestingly, the RBE value based on cell motility was consistent with that based on cell survival (i.e., ~4 versus 3.9). This finding provides important insight into treatment planning, i.e., the GTV–CTV margin can be set in a comparable manner for X-rays and carbon ions.

The bystander effect is a phenomenon whereby non-irradiated cells adjacent to irradiated cells are killed (43). Previous research shows that the bystander effect is universal among most types of normal cells and tumor cells (43). However, the significance of the bystander effect among different types of cells after carbon ion irradiation is not fully understood. Harada et al. investigated the bystander effect in carbon ion-irradiated A549 cells by using carbon ion microbeams (diameter = 20 μm) to irradiate only 0.0001–0.002% of the cells in a culture plate (44). The entire cell population was then subjected to a clonogenic survival assay, resulting in an 8–14% reduction in cell survival. Thus, the bystander effect plays a highly significant role in carbon ion-induced killing of A549 lung cancer cells. By contrast, Wakatsuki et al. found that the bystander effect played no role in the killing of a HTB-94 chondrosarcoma cell line (45). These data highlight the fact that different cell types show different susceptibilities to the bystander effect induced by carbon ion irradiation by up to ~10%. Further research into the carbon ion radiotherapy-induced bystander effects in different tumor cells and normal cells is necessary to optimize treatment planning.

## Research into Combination Therapy to Enhance the Efficacy of Carbon Ion Radiotherapy

Theoretically, a sufficiently high dose of ionizing radiation can sterilize any type of tumor (46). However, clinically applicable doses are delivered within a range that is tolerable by normal tissues (47). Dose escalation trials are underway to identify the maximum tolerable dose for carbon ion radiotherapy according to disease site (2). To date, clinical experience indicates that carbon ion radiotherapy can be delivered to many disease sites at higher biologically equivalent doses than 3D-CRT using X-rays. Carbon ion radiotherapy can also achieve nearly 100% tumor control probability in tumors that are uncontrollable by other radiation therapy modalities using X-rays and protons, such as spinal chordomas (1). Nonetheless, local recurrence occurs within the GTV, indicating the presence of a subset of carbon ion-resistant tumors.

To eradicate carbon ion-resistant tumors, it is important to establish an optimal form of combination treatment that increases the efficacy of carbon ion radiotherapy. To this end, several clinically available chemotherapeutic drugs have been tested in a preclinical setting. Kubo et al. examined the ability of carboplatin and paclitaxel to sensitize H460 lung cancer cells to carbon ion beams (48). Both sensitized cancer cells to carbon ion irradiation, with sensitizing ratios of 1.21 and 1.22, respectively (NB, a sensitizing ratio of >1 indicates that the radiation and the drug have a synergistic effect). These sensitizing ratios were comparable with those of X-rays. Similarly, Takahashi et al. demonstrated that etoposide sensitized X-ray-resistant rat yolk sac tumor cells to carbon ions, reporting a sensitizing ratio of ~1.2 (23). Carboplatin, paclitaxel, and etoposide are all currently used in combination with X-rays for clinical tumor treatment; carboplatin and paclitaxel are used to treat NSCLC, uterine cervical cancer, and esophageal cancer, and etoposide is used to treat small cell lung cancer. The combination of these drugs with carbon ions should likewise be tested in the clinic.

Several drugs currently under development have been tested for their ability to sensitize cells to carbon ion irradiation. Ma and colleagues examined the sensitizing effects of the Wee-1 inhibitor, MK-1775, using H1299 lung cancer cells (49). Wee-1 is a nuclear kinase protein involved in activating the G2 cell cycle checkpoint. Pretreatment for lung cancer cells with MK-1775 abrogated the induction of G2/M arrest after carbon ion irradiation, leading to an increase in mitotic catastrophe-mediated cell death. The sensitizing ratio of MK-1775 was 1.21 at 200 nM, a concentration at which MK-1775 alone reduces the surviving cell fraction by 50%. Musha et al. evaluated the sensitizing effect of the heat shock protein 90 (Hsp90) inhibitor, 17-AAG, in LMF4 oral squamous cell carcinoma cells (50). Hsp90 forms a chaperone complex with client proteins, thereby stabilizing them. Because various Hsp90 client proteins [e.g., Akt, ErbB2, and hypoxia-inducible factor-1α (HIF-1α)] are associated with malignant cancer phenotypes, Hsp90 is regarded as a potent molecular target (51–53). 17-AAG sensitized tumor cells to carbon ions with a sensitizing ratio of 1.14 at 100 nM, a concentration at which 17-AAG alone reduces the surviving cell fraction by 30–40%, although the underlying mechanism is unclear. These data indicate that Wee-1 or Hsp90 inhibition is a viable strategy for sensitization of carbon ions, but the sensitizing effect requires further testing in animal models. As a monomodality treatment for cancer, MK-1775 is currently under investigation in phase I and phase II clinical trials (54). Meanwhile, a phase II clinical trial for 17-AAG was terminated due to the lack of adequate tumor response and the presence of normal tissue toxicity (55). Nevertheless, a number of next-generation Hsp90 inhibitors are now being tested in multiple clinical trials (55).

Cancer immunotherapy has recently provoked a great deal of interest. Novel molecular targeting therapies (including those targeting programed cell death 1, programed cell death-ligand 1, and cytotoxic T-lymphocyte-associated protein 4) all demonstrate marked antitumor effects (56–58). Evidence suggests that the antitumor immune response plays an important role in the antitumor efficacy of X-ray radiotherapy. Nakano et al. showed that pretreatment levels of intratumoral infiltration by Langerhans cells and T cells, the key players in antitumor immune responses, correlates with a favorable outcome in patients with uterine cervical cancer treated using X-ray radiotherapy (59). They also showed that concomitant use of X-rays and intratumoral injection of sizofiran, an immune-response modifying drug, increases intratumoral infiltration of Langerhans cells and T-cells in patients with uterine cervical cancer (60). Recently, Suzuki et al. demonstrated that an antigen-specific T cell response is activated in esophageal cancer patients receiving combined X-ray radiotherapy and chemotherapy (61). These data suggest that the efficacy of X-ray radiotherapy can be improved upon combination with assorted cancer immunotherapies.

To investigate whether the antitumor immune response contributes to the antitumor efficacy of carbon ion radiotherapy, Yoshimoto et al. examined the impact of carbon ion irradiation on the release of high-mobility group box 1 protein (HMGB1) after irradiation in various cancer cell lines (62). HMGB1 is released from tumor cells damaged by radiotherapy and/or chemotherapy. Elevated serum HMGB1 levels are associated with activation of the antigen-specific T cell responses after chemoradiotherapy (61). The investigators found that HMGB1 levels in conditioned culture media were significantly higher after carbon ion irradiation. The RBE values based on HMGB1 release were similar to those based on clonogenic survival. These data suggest that the antitumor immune response contributes to an antitumor effect not only in X-ray radiotherapy but also in carbon ion radiotherapy. Additional preclinical research investigating the effects of combinations of carbon ion radiotherapy and cancer immunotherapy is currently underway.

## Perspectives

Carbon ion radiotherapy is a promising therapy for cancer. Appropriate patient selection based on individual tumor radiosensitivity is key to making the most of this medical resource with extremely limited availability. Recent advances in molecular biology research emphasize the need for functional predictive assays using tumor biopsy specimens for the practice of precision medicine (63). The utility of predictive assays for determining intratumoral oxygen levels, radiation-induced cellular apoptosis, DSB repair capacity, and gene mutational status should be tested in the clinic. Of note, recent studies demonstrate that a combination of distinct tumor features can work synergistically to predict prognosis in a subset of tumors, indicating the benefit of combined usage of these predictive assays (64). In addition, progress in the field of metabolomics indicates that non-invasive predictive assays based on biofluids, such as blood or urine, will be established in the near future (65, 66).

Researchers have accumulated extensive data concerning radiobiological properties of cancers and normal tissues. However, translation of biological data to the clinic remains far from satisfactory. This may be partially due the huge diversity in experimental systems used in radiation biology studies, making it difficult to draw solid conclusions for clinical applications. A meta-analytic approach to integrate the existing data is suggested. Moreover, specification of carbon ion beams including LET values employed in the studies must be carefully considered during the data translation process. Most *in vitro* studies used mono-energetic high-LET (i.e., ~100 keV/μm) carbon ion beams. However, several facilities, including NIRS and GHMC, now utilize spread-out Bragg peak (SOBP) carbon ion beams, which comprise a mixture of different LET beams, in the clinic. The biological effect of SOBP carbon ion beams likely differs from that of mono-energetic high-LET beams. Studies during the early era of carbon ion radiobiology provide plenty of data on the biological effect of SOBP carbon ion beams. Nevertheless, these data are difficult to interpret in the context of modern molecular biology and in a clinical setting because the biological effects were analyzed using biophysics models to deconvolute the mixed LET spectrum. Therefore, future studies should investigate the effects of SOBP carbon ion beams using current molecular biological techniques, particularly with respect to tumor hypoxia, radiation-induced apoptosis, and DSB repair.

Emerging molecular biology techniques are expected to contribute to further advancement of translational research in carbon ion radiobiology. First, next-generation sequencing technologies will almost certainly identify specific genomic and epigenomic profiles that affect radiosensitivity (28, 67) and can be combined with existing data concerning expression profiles related to radiosensitivity (68, 69). Second, advanced high-resolution microscopy techniques will clarify the molecular processes that occur following carbon ion irradiation. For example, Britton et al. visualized recruitment of a single Ku molecule at DSB sites, which is essential for the repair of DSBs induced by ionizing irradiation (70). Thus, advanced high-resolution microscopy will promote our understanding of the repair kinetics of complex DSBs induced by carbon ions. Third, emerging imaging technologies will enable detailed visualization of intratumoral oxygen levels and metabolomic states (71). We anticipate that integration and translation of data in radiation biology will greatly improve the efficacy of carbon ion radiotherapy.

## Author Contributions

TO, HS and S-eN summarized data and drafted the manuscript. TN supervised the manuscript. All authors read and approved the final manuscript.

## Conflict of Interest Statement

The authors declare that the research was conducted in the absence of any commercial or financial relationships that could be construed as a potential conflict of interest.
